# Long term trends in control of hypertension in the Northern Sweden MONICA study 1986–2009

**DOI:** 10.1186/s12889-015-2280-6

**Published:** 2015-09-24

**Authors:** Ellinor Törmä, Bo Carlberg, Marie Eriksson, Jan-Håkan Jansson, Mats Eliasson

**Affiliations:** Department of Public Health and Clinical Medicine, Sunderby Research Unit, Umeå University, Umeå, Sweden; Department of Public Health and Clinical Medicine, Medicine, Umeå University, Umeå, Sweden; Department of Statistics, Umeå School of Business and Economics, Umeå University, Umeå, Sweden; Department of Public Health and Clinical Medicine, Research Unit Skellefteå, Umeå University, Umeå, Sweden

**Keywords:** Cohort blood pressure hypertension trend

## Abstract

**Background:**

A large proportion of treated hypertensive subjects do not achieve target blood pressure (BP) levels. We investigated trends in treatment and BP levels in the population, and among treated hypertensive subjects in northern Sweden.

**Methods:**

The six Northern Sweden MONICA population surveys 1986 to 2009, included 6342 subjects aged 45 to 74 years of age, participation rate 79.3 %. Factors associated with lack of BP control are presented for 1106 participants in 2009. BP control was defined as a systolic BP <140 and a diastolic BP <90 mm Hg among treated hypertensive patients.

**Results:**

Between 1986 and 2009, the proportion of the population that received antihypertensive treatment increased. The proportion of the whole population having BP <140/90 mm Hg increased for all (*p* < 0.001 for each subgroup), except for men 45–55 years old. In 2009, 62.4 % of the population had BP <140/90 mm Hg, 67.2 % in women and 58.1 % in men (*p* = 0.002). In the group of treated hypertensive patients, the proportion having BP control increased (*p* < 0.001) with no difference between sex or age groups. In 2009 52.1 % of treated hypertensives had BP control.

In 2009, adequate BP control among treated hypertensive patients was 63.9 % for those with BMI <25, but only 48.8 % for those with BMI > 25 (*p* = 0.015). Abdominal obesity was associated with less BP control (48.1 %) than without abdominal obesity (66.2 %, *p* = 0.007). Women who were physically inactive had better BP control than those who were active (*p* = 0.03). Men treated with two or more antihypertensive drugs were 50 % more likely to reach target BP than men with monotherapy (60.4 % vs. 40.0 %, *p* = 0.035). Rural or urban living, level of education, diabetes mellitus or having a high cardiovascular risk were not associated with better BP control.

**Conclusion:**

Antihypertensive treatment and BP control have increased in northern Sweden since 1986, although in 2009 still barely half of the treated patients achieved adequate BP levels. Intensified treatment and weight reduction may help to further improve BP control.

## Background

High blood pressure (BP) is estimated to cause 9.4 million premature deaths throughout the world each year and has the highest attributable mortality of cardiovascular risk factors [1]. It almost triples the risk for all subtypes of stroke and myocardial infarction [2]. An increase of 20 mm Hg in systolic, or 10 mm Hg in diastolic BP, doubles the risk of dying from stroke or ischemic heart disease [3]. Although hypertension is largely amendable through adequate antihypertensive treatment [4], more than two thirds of the treated hypertensive population in the world continue to have BP levels above the target limit [5].

According to the 2013 guidelines from the European Society for Hypertension and the European Society for Cardiology, treatment should be considered for subjects at high cardiovascular risk, until the BP is below 140/90 mmHg [6]. During the 1990s, 54 % of treated hypertensive patients in the US and only 19 to 40 % in Europe had controlled BP (defined as BP <140/90 mm Hg) [7]. Only 32 % of treated hypertensives worldwide between 35 and 70 years reached this target BP [5].

The multinational MONICA study between 1986 and 1994 compared hypertension control between 24 areas and found that subjects treated for hypertension in northern Sweden were among those with the lowest prevalence of control [8]. In the county of Västerbotten in northern Sweden, treatment increased in the population between 1990 and 2010, and control among treated increased to 65 % in 2009 [9]. Sex, education level and number of drugs for hypertension are among factors proposed to determine control rates [7, 9–12].

Reliable and recent data on BP control from well standardised and validated population studies are lacking. Because use of anti-hypertensive medication increased markedly in northern Sweden in the mid-1990s [13], we aimed to describe how, and if, this influenced the control of hypertension between 1986 and 2009. We also investigated factors possibly associated with lack of control among the treated patients today and looked for factors that might be amendable to the health care system or lifestyle changes.

## Methods

Our analysis is based on the six surveys in the Northern Sweden MONICA study. The first survey took place in the early spring of 1986, and the following five surveys were conducted at the same time of the year, and the most recent took place in 2009. Similar procedures for sampling, anthropometric measurements and blood pressure were used throughout the whole study.

### Participants

Details of sampling and selection have been presented previously [14]. The participants were randomly selected from population registers in the two most northern counties of Sweden (target population 312 000) [13]. For each survey the subjects were age-stratified into 10-year age groups (25 to 74 years) with a total of 250 men and women in each stratum. In this analysis, only subjects between 45 and 74 years of age were included due to the low prevalence of treated hypertensive patients among the younger participants. For the 1986 and 1990 surveys only data for subjects 45 to 64 years were available. Through telephone interviews we evaluated non-responder bias [14]. In the first three surveys, non-responders were more likely to be obese or hypertensive, more likely to smoke, but had a similar educational level. In 2009 non-participants were younger, had lower education and more often were smokers or diabetics.

The study was approved by the Research Ethics Committee of Umea University, and all subjects gave informed consent to participate.

### Measurement procedures

Details regarding survey procedures have been published [14]. The teams conducting the examinations were specially trained by the same instructors to ensure correctness and consistency. Information regarding life style, social and medical history were extracted from material collected by survey questionnaires. BP was measured twice in sitting position, after a 5-min rest, using the Hawksley random-zero sphygmomanometer, the mean value was recorded [15].

Control among treated hypertensive subjects was defined as having a systolic BP of <140 and a diastolic BP <90 mm Hg. Current antihypertensive drug treatment was determined by asking: “Are you taking (in the last 2 weeks) drugs for high BP?” [14]. Physical activity was defined as exercising on a regular basis or not. “Regular exercise” ranges from light exercise twice a week to high intensity/vigorous workout several times a week. “No regular exercise” included subjects performing occasional low-intensity exercise or not exercising at all. Urban or rural living was determined by the population size of the community; >15 000 inhabitants was considered to be urban and ≤15 000 rural. “Educational level” was defined as having attended university or not.

Normal weight was defined as BMI <25, overweight as BMI 25–29,9, and obesity as BMI of 30 or aboveAbdominal obesity was defined as a waist circumference of 94 cm or more for men, 80 cm or more for women. High cardiovascular risk was defined by self-reported previous stroke, myocardial infarction, coronary surgery, or having diabetes. Onehundred and sixty nine of the 6229 subjects had high cardiovascular risk (15.2 %). Number of antihypertensive drugs was defined as using 1 or >1 BP-lowering drug(s), based on self-reported information.

Predictors and factors associated with lack of BP control are only presented for the 2009 participants.

### Statistical methods

Data are presented as means or proportions with 95 % confidence intervals (CI). Time trends for categorial variables were analyzed by Chi-square test for linear trends. Univariable och multivariable logistic regression was used to analyze the association between age, sex and other factors potentially associated with hypertension control. Two-by-two interaction terms including age group or sex were added to the regression model to study if effects of other factors varied by age or sex.

## Results

In the six MONICA population surveys between 1986 and 2009, 8000 subjects between 45 and 74 years of age were invited. 6342 subjects (79.3 %) participated, 50.1 % women and 49.9 % men. BP values were missing in six subjects and data on treatment with BP lowering drugs were missing in 108 subjects leaving 6229 subjects included in the statistical analyses. For 44 subjects treated with BP lowering drugs, the exact numbers and type of drugs were not known.

### Trends in treatment and control 1986 to 2009

**Fig. 1:**
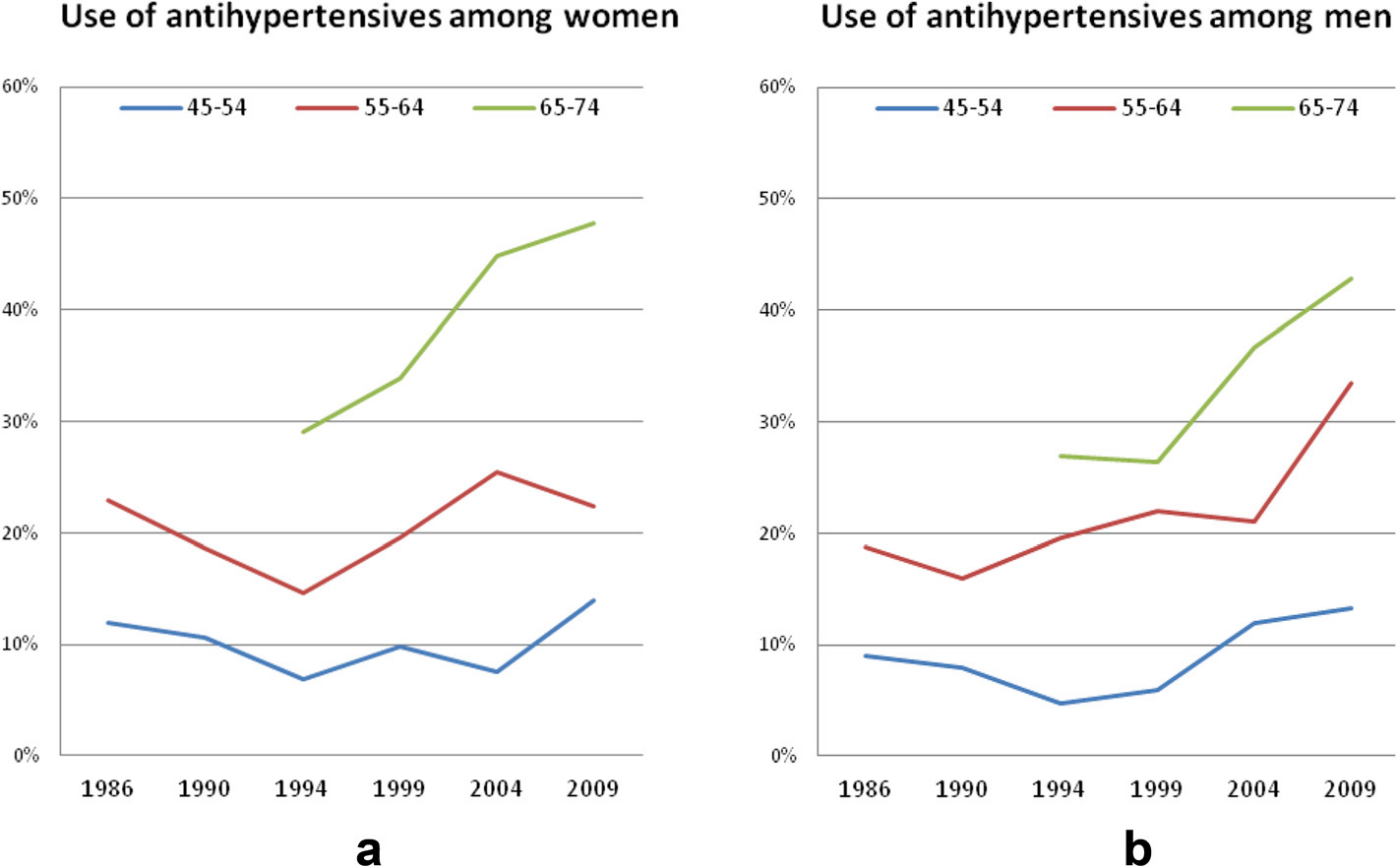
Time trend in prevalence of treatment 1986 to 2009, by gender (**a**: women, **b**: men) and age group

From 1986 to 1994, the use of BP lowering drugs remained steady or even decreased in some age groups, but the use subsequently increased (*p* <0.001 for the whole population 45 to 74 years) (Fig. 1). The tendency was similar for men and women and in the different age groups with no interaction between sex or age group and year of survey.

**Fig. 2:**
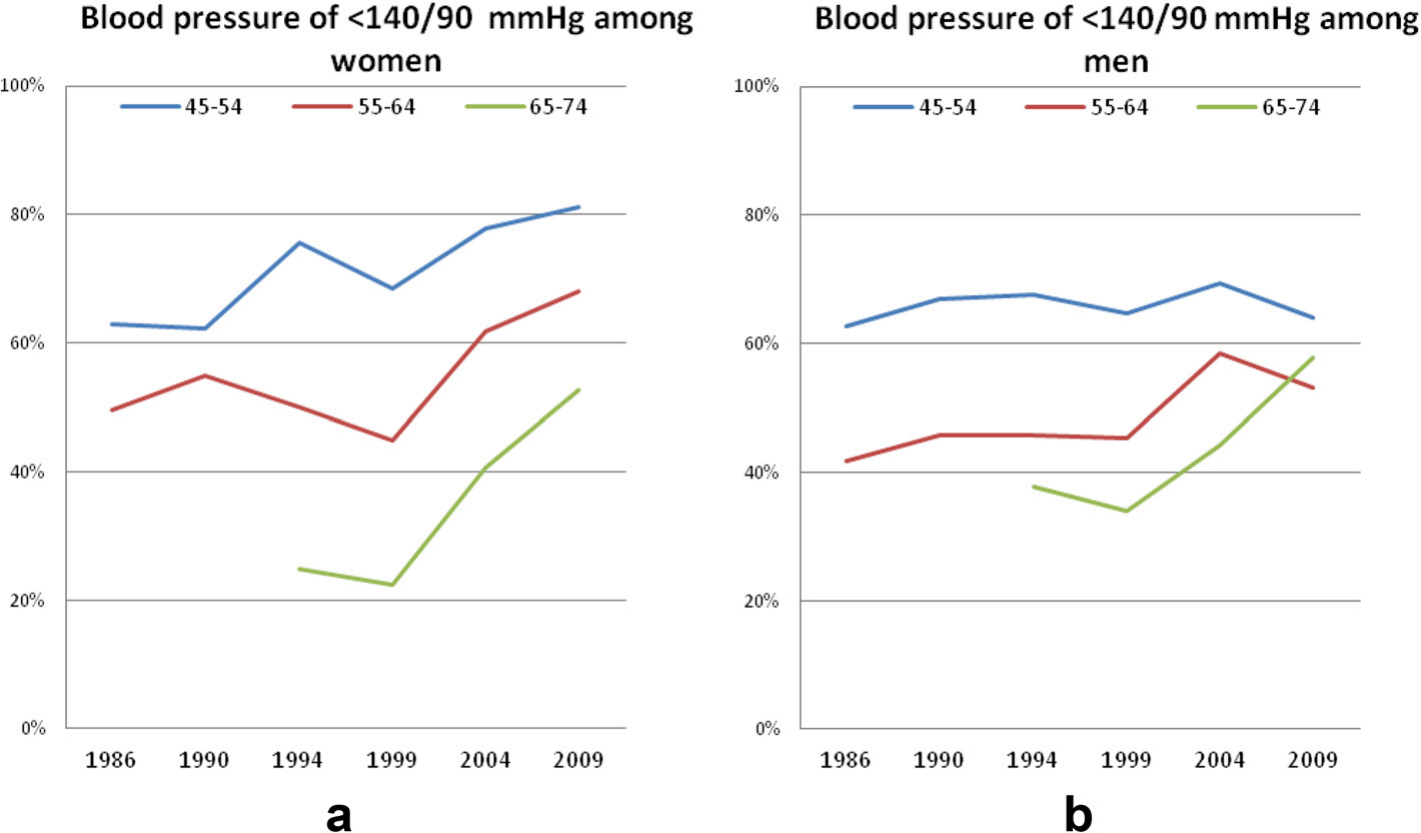
Proportions of subjects with BP <140/90 in the population and time trends from 1986 to 2009, by gender (**a**: women, **b**: men) and age group

Between 1986 and 2009, the proportion having BP control (<140/90 mm Hg) increased in the whole population (p *< 0.001)*. The effect differed by age group (*p* <0.001) but not by sex (Fig. 2). The increase was evident in all age and sex strata except for the youngest men (*p* < 0.001).

In 2009, BP control among subjects 45–74 years was 62.4 %, and more prevalent in women (67.2 %) than in men (58.1 % *p* = 0.002) (Table 1).

**Table 1:** Sample sizes and prevalences of hypertension, treatment and control in 2009

Variable	Sex	Age group (years)	n, % (within age group)
Participants	Women	45–54	177
		55–64	188
		65–74	185
		Total (45–74)	550
	Men	45–54	169
		55–64	190
		65–74	197
		Total (45–74)	556
Treatment in the population	Women	45–54	14.0
		55–64	22.4
		65–74	47.8
		Total (45–74)	28.1
	Men	45–54	13.3
		55–64	33.5
		65–74	42.9
		Total (45–74)	30.6
Control in the population (SBP < 140 and DBP < 90 mm Hg)	Women	45–54	81.2
		55–64	68.1
		65–74	52.7
		Total (45–74)	67.2
	Men	45–54	64.1
		55–64	53.2
		65–74	57.9
		Total (45–74)	58.1
Control among treated (SBP < 140 and DBP < 90 mm Hg among treated)	Women	45–54	58.3
		55–64	58.5
		65–74	45.9
		Total (45–74)	51.3
	Men	45–54	50.0
		55–64	48.3
		65–74	56.8
		Total (45–74)	52.8

**Fig. 3:**
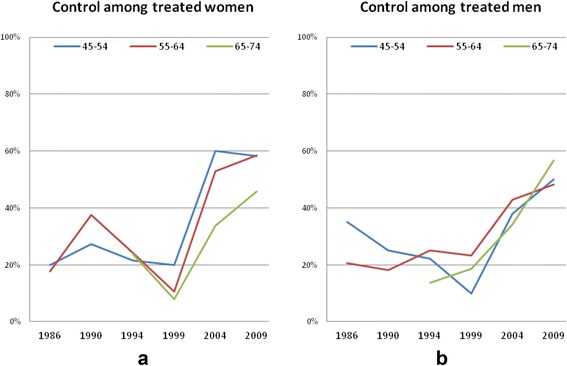
Time trend in BP control among subjects treated 1986 to 2009, by gender (**a**: women, **b**: men) and age group

After an initial decrease, BP control increased rapidly in the whole group of treated hypertensive subjects (*p* < 0.001) (Fig. 3). This trend did not differ by sex or age group.

### Factors associated with inadequate BP control in 2009

In the 2009 MONICA survey, 1106 subjects aged 45–74 years participated with fairly equal numbers in each age stratum and between genders (Table 1). Fifteen hundred subjects were invited and thus the participation rate was 73.7 %. Treatment with BP lowering drugs in the population was 29 %, with no difference betweeen men and women. An average of 1.77 BP lowering drugs was used. The most commonly used antihypertensive drugs acted on the RAA-system (used by 59.5 %), followed by beta-adrenergic blockers (52.4 %), diuretics (including tiazides, loop diuretics and spironolactone) (38.3 %) and calcium channel blockers (27.3 %).

Among the 313 treated hypertensive subjects, 52.1 % had adequate BP control.

The difference in BP control between men and women was negligible, 52.8 % men vs. 51.3 % women, and there was no differerence by age group (Table 1).

One fifth (20.3 %) of the population aged 45–74 years was physically inactive in 2009. Almost a quarter of the subjects treated with BP lowering drugs were inactive. Physically active women had less BP control than physically inactive women (46.0 vs. 66.7 %, *p* = 0.03). There was no association between degree of physical activity and BP control among men. A test for interaction confirmed that the effect differed by sex (*p =* 0.028). There was a fairly equal distribution of subjects living in rural vs. urban communities, and BP control did not differ between them.

In 2009, 16 % of the population aged 45–74 years used one anti-hypertensive drug and 11 % used two or more. In the treated patients, approximately 40 % used two or more drugs. Treatment with a combination of BP lowering drugs was more common among older patients. Among all treated hypertensives, treatment with one vs. two drugs or more was not associated with any difference in BP control, 48.3 vs. 55.4 % (*p =* 0.3). Among women no difference was seen, but among men the corresponding figures were 40.0 % and 60.4 %, respectively (*p* = 0.035).

Three quarters of the subjects had not attended university and were defined as having a lower level of education, and that proportion was similar in subjects with or without treatment for hypertension. There were no differences in BP control according to level of education among hypertensive subjects. BP control did not differ between subjects with or without diabetes, 63.0 vs. 49.6 % (*p* = 0.07).

Among the entire 6229 subjects, 22 % were obese and 43 % were overweight. The use of BP lowering drugs increased with BMI, and among men and women with obesity, nearly 50 % used antihypertensives. BP control did not differ across the three BMI categories. When merging the overweight and obese groups, BP control was found in 64 % of the normal weight subjects, but in only 49 % of the overweight + obese group (*p* = 0.024).

Almost two thirds had abdominal obesity, more so among subjects treated with antihypertensives (59.2 vs. 77.2 %). Only 48.1 % of the subjects with abdominal obesity had BP control, whereas the corresponding figure for those without abdominal obesity was 66.2 % (*p* = 0.007).

Among the entire 6229 subjects, 15.2 % were classified as having a high cardiovascular risk, defined as having had a previous stroke, myocardial infarction, coronary surgery, or having self-reported diabetes. Among those treated with BP lowering drugs, 30.3 % had a high cardiovascular risk. There was no association between BP control and having a high cardiovascular risk.

When adjusting for age (as a continous variable) and sex in a logistic regression, none of the variables -- physical inactivity, rural living, number of BP lowering drugs, level of education or high cardiovascular risk -- were associated with BP control. Overweight and obesity were associated with less BP control (*p* = 0.023, OR 1.89, 95 % CI 1.09; 3.25), and abdominal obesity was also associated with less BP control (*p* = 0.008, OR 2.1, 95 % CI 1.2; 3.7).

## Discussion

Antihypertensive treatment increased in northern Sweden between 1986 and 2009, which may partly explain why the proportion having BP control (BP <140/90) increased both in the population and among the treated hypertensive subjects. Since almost half of the treated subjects did not reach target levels we searched for characteristics that would be amendable to the health care system or to lifestyle changes. Inadequate BP control in 2009 was not signficantly associated with gender or age, but it was associated with overweight or general and abdominal obesity. In men, using two or more antihypertensive drugs was associated with better BP control. Other socioeconomic, life style and treatment-related factors did not contribute to better control, although the low numbers in these subgroups should caution against strong conclusions.

Trends toward increased BP treatment were seen in almost all groups, most noticeably after 1994, as previously reported [13]. Similar trends are reported from the US [16]. In 2009, the use of antihypertensive medication in the older population in northern Sweden was somewhat less common (Sweden 45 %) than in Canada (52 %) [12]. Recent studies in Europe report treatment rates in the population >20 years of age around 10 % to just above 40 % [9, 10, 17–19], which are comparable to our 30 % treatment level.

Until the mid 1990s, many Swedish physicians were reluctant to treat otherwise healthy individuals with BP lowering drugs. Early results from the MONICA project showed a higher rate of cardiovascular events in northern Sweden than in many other western European countries, which probably lead to a change in attitude and an intensification of antihypertensive treatment in the mid-1990s in Sweden [20, 21]. The publication from the Swedish Health Technology Assessment Agency in 1994, Moderately Elevated BP, updated in 2004 [22], emphazised risk stratification and the use of more than one drug. Therefore a greater focus was put on adequate case finding, life style advice and treatment to target levels.

BP trends in the whole population mirror the effects of medical treatment of some subjects and possibly also the influence of factors in the society such as increasing obesity, changes in diet and changes in physical activity. In 2000, BP control in the population in northern Sweden was less than in the USA [16]. A Swiss study reported 73 % BP control in 2009 compared with 63 % the same year in MONICA [23]. A Canadian study found 79 % BP control among 60–79 years olds in 2009, which was better than the 55 % among elderly in northern Sweden in 2009 [12]. Thus, a concerted action to lower BP in the general population of Sweden with both life style changes and drug treatment is warranted.

Trends towards better control among treated hypertensive patients were seen in several countries. In this study, BP control increased rapidly and reached 52 % in 2009. That was greater than that reported in Lithuania and Denmark [10, 24], similar to that reported in the US 2000 and Italy in 2012, but below that found in Switzerland 2009 (59 %) [16, 17, 23] and far lower than that reported in Canada in 2009 (73 % 60–79 years) [12].

Sex had no effect on BP control in our study, and results in the literature vary. Some studies show women are more likely to have BP control [7, 9, 10, 23], while others find that men are more likely to have BP control [8, 12, 16].

Age would be expected to increase blood pressure because blood vessels become stiffer and glomerular filtration rate decreases with age, which would affect the response to treatment [6, 25, 26]. BP-lowering drugs are less well tolerated among older subjects, which leads to a more restrictive attitude from physicians towards treatment and BP goals [27]. However, we found no effect of age on BP control. Some studies have found no effect of age [23], while others have found better BP control in the elderly [12, 28, 29]. Among those reporting less BP control among older subjects, it seems like elderly women have less BP control than men [12, 16].

Self reported physical activity was lower among women with good BP control than women with poor BP control in our study. Perhaps women with too little effect from their BP treatment or with refractory hypertension purposely exercise more to try to improve their BP control. Similar results were recently reported from Canada [12], but in Switzerland and Spain a sedentary lifestyle was associated with less satisfactory BP control [23, 30].

Large areas in northern Sweden are sparsely populated, and the long distances might lead to difficulties to obtain healthcare, which could lead to infrequent contact with health care personnel, poor detection of illness and poor follow-up of disorders such as hypertension. Even so, we found no association between BP control and size of communityThis indicates that health care in northern Sweden manages to provide care of equal quality regardless of living conditions for people with hypertension, and this is corroborated by a recent study in Poland [28].

Multiple antihypertensive drugs in men induced better BP control than monotherapy in our study, but only a minority used two or more drugs. In contrast, another Swedish study found better BP control among people treated with monotherapy [31]. Perhaps patients with multiple antihypertensive drugs have a more severe and/or refractory hypertension, *i.e.* confounding by indication in non-randomized cohort studies. Recently published randomized control trials support our findings, present similar advantages with combination therapy, and emphasize the benefit from initial combination therapy, as the effect might be reduced if initiated later on [32]. Physicians might have an over-reliance on monotherapy, or reluctance towards combination therapy, which could explain why hypertensives continue to receive monotherapy despite inadequate BP control [11]. As many as 95 % of people with hypertension might be able to reach target BP, mainly through combination therapy [4].

Socioeconomic characteristics determine many traits of disease and response to treatment, but we found no association between educational level, as a proxy for socioeconomic status, and BP control. Some studies show no effect of educational level on BP controll whereas others show somewhat better BP control with higher education [9, 12, 23, 28, 33].

The aetiology behind type-2 diabetes and the cardiovascular and renal complications that follow type-2 diabetes, may reduce the efficacy of treatment among older diabetics and make it more difficult to achieve BP control. Some studies show no association between having diabetes and BP control [18, 23, 34], whereas others show poorer BP control among diabetics [35]. Since hypertension in diabetics increases the risk of both cardiovascular disease and end stage renal diseases, it is disappointing that BP control is so often lacking among diabetics.

Obesity and overweight were associated with poorer BP control than normal weight. Arterial stiffness, which is a consequence of diet-induced obesity, precedes and may contribute to the development of hypertension [36]. This association may explain why arterial stiffness is not found to the same extent among people with essential hypertension, as among those with obesity, isolated systolic hypertension, chronic kidney disease and old age [37]. Results similar to ours were observed in a study from Poland [28] but not in Swizerland or Canada [12, 23] where control rates were higher than in northern Sweden. This indicates a more offensive approach when treating hypertensive patients until reaching goal BP. This may especially benefit people with obesity.

Abdominal obesity was associated with markedly decreased BP control in our study. In a Chinese study, BMI, and not abdominal obesity, was more related to poor BP controll [38]. Endocrine secretion differs between intra-abdominal and subcutaneous fat [39]. Adipokines, bioactive peptides and proteins released from intra-abdominal fat, are fundametal in the pathogenesis of the metabolic syndrome and probably also in atherosclerosis. Intra-abdominal adipose tissue secrets high levels of a precursor to angiotensin II, which is known to cause BP elevation. Specific properties of intra-bdominal fat may block the effect of antihypertensive drugs and contribute to hypertension. Therefore, abdominal obesity is an independent predictor of resistant hypertension [40]. Abdominal obesity was the only predictor regarding deficient control that was found among both men (second largest impact of the factors observed) and women (largest impact) in an elderly population of Stockholm [31]. Studies on abdominal obesity as a predictor of BP control are scarce, and futher research is merited due to its strong impact.

We found no association between having a high cardiovascular risk and hypertension control. It is desirable that a substantially larger proportion of people with high cardiovascular risk achieve target BP levels to avoid further cardiovascular events, since lowering BP might be of greatest absolute benefit for them [35]*.* In other studies poorer BP control rates were found among hypertensive patientes with cardiovascular diseases [35], whereas previous myocardial infarction and/or angina pectoris were associated with increased BP control rates [31].

A major strength In the Northern Sweden MONICA study is its strict and uniform methodology throughout the whole time period from 1986 to 2009. BP readings by random-zero methods avoid digit preference and anthropometry measurements were made by trained staff using similar equipment and protocols. Comparisons between BP measurements with random-zero and an automated oscillometric device, show higher accuracy in favour of random-zero measurement methods [15].

Note that patients with white-coat hypertension were not correctly diagnosed using the mean of 2 BP mesurements. However, the aim of this study was not to diagnose clinical hypertension. We aimed to describe an epidemiological picture of hypertension in the population, using methods common with all major population studies. Another limitation is that information given in a questionnare can be inaccurate and self-reported physical activity was probably the least reliable variable. In addition, there has been a declining participation rate in the Northern Sweden MONICA Study, from 81 % to 69 % over the study period, and this is almost entirely due to the loss of younger men and women [14]. In the age groups now studied we had a high participation rate, 79.3 %, which supports the validity of our data. Non-significant effects may be due to the relatively low sample size in some sub groups.

## Conclusions

In conclusion, antihypertensive treatment increased in northern Sweden between 1986 and 2009, most evident among the elderly. A probable consequence of that is that BP control has increased in the population and among treated hypertensives. Even so, further effort is needed since several studies show that it is possible to achieve far higher BP control rates. The health care systems are similar in Sweden and Canada, and both are mainly tax funded [41]. In Canada, more than 90 % of the hypertensive patients are treated and 80 % reach the BP goal. It should be possible for Sweden to reach the same treatment goals [12].

Overweight, obesity, and abdominal obesity are all associated with lack of BP control, thus making these groups a target for intensified antihypertensive therapy. If BP goals are not achieved promptly, it is reasonable to try treatment with one or more additional antihypertensive drugs, since it is possible to achieve a higher prevalence of control with combination therapy [4].
